# *Chlamydomonas agloeformis* from the Ecuadorian Highlands: Nutrients and Bioactive Compounds Profiling and In Vitro Antioxidant Activity

**DOI:** 10.3390/foods12173147

**Published:** 2023-08-22

**Authors:** Teresa Grande, Andrea Vornoli, Valter Lubrano, Francesco Vizzarri, Andrea Raffaelli, Morena Gabriele, Jeniffer Novoa, Carla Sandoval, Vincenzo Longo, Maria Cristina Echeverria, Luisa Pozzo

**Affiliations:** 1 Institute of Agricultural Biology and Biotechnology-National Research Council (IBBA-CNR), Via Moruzzi 1, 56124 Pisa, Italy; teresa.grande@unifi.it (T.G.); andrea.vornoli@ibba.cnr.it (A.V.); andrea1.raffaelli@santannapisa.it (A.R.); morena.gabriele@ibba.cnr.it (M.G.); vincenzo.longo@ibba.cnr.it (V.L.); 2 Department of Experimental and Clinical Biomedical Sciences “Mario Serio”, University of Florence, Viale Morgagni 50, 50134 Florence, Italy; 3 Fondazione G. Monasterio, CNR/Regione Toscana, 56124 Pisa, Italy; walterl@ftgm.it; 4 National Agricultural and Food Centre Nitra, Hlohovecká 2, 95141 Lužianky, Slovakia; francesco.vizzarri@nppc.sk; 5 Crop Science Research Center, Scuola Superiore Sant’Anna, Piazza Martiri della Libertà 33, 56127 Pisa, Italy; 6 eCIER Research Group, Department of Biotechnology, Universidad Técnica del Norte, Av. 17 de Julio 5–21 y Gral. José María Córdova, Ibarra 100150, Ecuador; jpnovoar@utn.edu.ec (J.N.); casandoval@utn.edu.ec (C.S.); mecheverria@utn.edu.ec (M.C.E.)

**Keywords:** green microalgae, antioxidant, Human Microvascular Endothelial Cells (HMEC-1), functional food

## Abstract

Green microalgae are single-celled eukaryotic organisms that, in recent years, are becoming increasingly important in the nutraceutical, cosmetic, and pharmaceutical fields because of their high content of bioactive compounds. In this study, a particular green microalga was isolated from freshwater highland lakes of Ecuador and morphologically and molecularly identified as *Chlamydomonas agloeformis* (ChA), and it was studied for nutritional and nutraceutical properties. The phenolic composition and the fatty acids profile of lyophilized cells were determined. The methanolic extract was analyzed for the phenolic compounds profile and the antioxidant capacity by means of in vitro tests. Finally, Human Microvascular Endothelial Cells (HMEC-1) were exploited to explore the capacity of ChA to reduce the endothelial damage induced by oxidized LDL-mediated oxidative stress. The extract showed a good antioxidant ability thanks to the high content in polyphenolic compounds. The observed decrease in HMEC-1 cells endothelial damage also was probably due to the antioxidant compounds present in the extract. Based on the outcomes of our in vitro assays, ChA demonstrated to be a promising source of bioactive compounds possessing exceptional antioxidant capacities which make it a prospective functional food.

## 1. Introduction

Microalgae are gaining increasing attention in scientific research as a promising solution to many environmental and economic challenges. They can represent a useful tool to promote the change in people’s eating habits both to improve health and reduce the environmental impact due to the high population growth rates and the resultant demands for essential nutrients [1].

It has been estimated that there are 800,000 species of microalgae and that only 5–10% of these are known [2]. Green microalgae represent one of the most numerous classes to which various genera and species belong, and only a small part of them has been studied for their potential as nutraceuticals. They are widespread all over the world, but areas with great biodiversity, such as Ecuador, represent a more promising resource for the isolation of new species rich in new bioactive substances [3]. The country’s warm and humid climate, along with its coastal and inland water bodies, provide suitable conditions for the growth and proliferation of green microalgae, which are known for their high nutritional value and are considered a potential source of food for both humans and animals. These photosynthetic microorganisms represent a sustainable supply of numerous nutrients with important attributes, like proteins, ω-3 fatty acids, carotenoids, vitamins, and minerals [4]. Indeed, they are able to convert inorganic substances into natural organic compounds that can be used as functional ingredients with antimicrobial, anti-inflammatory, antiallergic, antioxidant, hypoglycemic, and immunosuppressive properties [5]. Many of these compounds are represented by their metabolic by-products generated during their cell cycle. Among them, phenolic compounds represent one of the most interesting classes of bioactive compounds [6]. To date, fruits and plants have represented the principal source of natural polyphenols available on the market, but microalgae are becoming increasingly important. Some studies have shown that the phenolic compounds content contributes to the remarkable antioxidant capacity typical of microalgae [7,8,9]. In various diseases like diabetes, hypertension, and dyslipidemia, microcirculation assumes a crucial role in the progression of damage to target organs. Animal models affected by metabolic syndrome exhibit changes in the structure of microvessels, including reduced lumen size, thinner vascular walls and atrophic remodeling. These alterations take place before the onset of diabetes and in the absence of atherosclerotic lesions [10]. Furthermore, there is a significant reduction in microvessel density, which negatively impacts the transportation and exchange of substances with parenchymal tissues [11]. This compromised vascular response may limit the perfusion of peripheral tissues. In metabolic diseases, the microcirculatory system is the first to be impacted when compared to larger blood vessels. Furthermore, among the various types of endothelial cells in the human body, microcirculatory endothelial cells are the most abundant and representative. For these reasons, Human Microvascular Endothelial Cells (HMEC)-1 are suitable to explore the potential of a microalga in mitigating endothelial damage induced by oxidative stress from stressors. A recent study by Le Goff et al. [12] showed that the extract of the green microalga *Ostreococcus Tauri* can influence the Benzo[a]Pyrene-induced toxicity in HMEC-1 cells. Indeed, cell death by apoptosis and the release of extracellular vesicles decreased, most probably due to the downregulation of CYP1A1, an enzyme involved in the activation of B[a]P. Moreover, the expression of inflammatory cytokines IL-8 and IL1-β induced by B[a]P was also reduced.

*Chlamydomonas agloeformis* (ChA) is a species of unicellular green microalgae, belonging to *Chlorofite* class. At present, Chlamydomonas sp. does not have approval for use in food applications within the European Union and only one species, *Chlamydomonas reinhardtii*, is recognized by Food and Drug Administration as GRAS (Generally Recognized As Safe) for human consumption [13]. The first aim of the present work was to isolate and morphologically and molecularly characterize the green microalga ChA. ChA powder was then evaluated for the proximate and fatty acids composition, the phenolic compounds content, and the antioxidant capacity of the methanolic extract. Finally, we analyzed the protective effects of ChA on the functional properties of HMEC-1 exposed to Oxidized Low-Density Lipoprotein (Ox-LDL) as a source of oxidative stress.

## 2. Materials and Methods

### 2.1. Chemical and Reagents

All reagents, media, and medium supplements used for cell culture were obtained from Sigma-Aldrich (St. Louis, MO, USA).

### 2.2. Microalgae Isolation and Cultivation

Sampling was carried out in Yahuarcocha lake, located in the province of Imbabura, Ecuador, at a depth of 0.50 m using a Van Dorn bottle and phytoplankton mesh (20 µm). To avoid light damage in the collected microalgae, all bottles were stored in darkness [14]. Microalgae was isolated using different solid and liquid culture media to determine the optimal nutritional condition for cell adaptation. The media were based on the following concentrations: 0.05 g/L CaCl_2_·2H_2_O, 0.05 g/L FeSO_4_·7H_2_O, 0.2 g/L MgSO_4_·7H_2_O, 0.005 g/L MnSO_4_·H_2_O, 0.002 g/L ZnSO_4_·7H_2_O, 0.002 g/L CuSO_4_·5H_2_O, 0.0006 g/L H_3_O_3_, 0.0001 g/L Na_4_MoO_4_, 0.0001 g/L CoCl_2_. KNO_3_ was used as a nitrogen source in different concentrations (1, 3, 5 g/L), and K_2_HPO_4_ as a phosphate source (0.5 and 0.7 g/L). Additionally, Murashige and Skoog (Duchefa Biochemie, BH Haarlem, the Netherlands) medium (4.4 g/L) was used with extra nitrogen source as KNO_3_ (2.0 g/L) and phosphate (0.5 g/L) [15]. All media were prepared with 0.3 g/L of NaHCO_3_, adjusted at pH 7, and autoclaved for sterilization. For the liquid samples, they were purified by serial dilutions and plate streaking. All samples were filtered using Whatman filters (10 µm). The part of the sample that remained in the filter was inoculated in Petri dishes with solid media. All liquid media and Petri dishes were incubated at 26 °C with 24:0 light/dark photoperiod, and alight intensity of 100 ± 10 µmol/m^−2^ s^−1^ for 21 days.

### 2.3. Morphological and Molecular Identification

The genomic DNA was extracted from the fresh algal isolated using the PureLink^®^ Plant Total DNA Purification Kit (Invitrogen, Waltham, MA, USA). Master Mix PCR Taq with dye (amb^®^) was used to PCR amplification, using the forward primer p23SrV-f1 (50-GGA CAG AAA GAC CCT ATG AA-30) and reverse primer p23Sr-r1 (50-TCA GCC TGT TAT CCC TAG AG30) [16]. Additionally, other segments were amplified using the primers ITS1 (5′-TCCGTAGGTGAACCTGCGG-3′) and ITS4 (5′-TCCTCCGCTTATTGATATGC-3′) [17]. Electrophoresis was performed to analyze PCR product on (1.5%) agarose gels adding 5 µL Sybr safe (Thermo Fisher Scientific, Waltham, MA, USA) in 80 mL of TBE 1x buffer. Lastly, the genetic identification was carried out using Basic Local Alignment Search Tool (BLAST) of National Center for Biotechnology Information (NCBI). The isolated strain was identified at the species level, and the corresponding sequences were deposited in GenBank. To confirm the results obtained, molecular identification was performed twice

### 2.4. Centesimal Composition and Fatty Acids Profile 

Ground samples of ChA green algae (<1 mm) were subjected to analysis for various components, including dry matter (DM, method 950.46), ash (method 920.153), lipid (method 920.39), crude protein (method 990.03), and crude fiber (method 978.10). These analyses were conducted following the standard methods outlined by the Association of Official Analytical Chemists [18], and results are expressed as % on dry weight. To determine the composition of fatty acids (FAs) in ChA, gas chromatography was employed after the process of extraction and methylation [19,20]. The gas chromatography analyses with flame-ionization detection (FID) were carried out using an Agilent 7890A chromatograph equipped with a chromatography column specifically designed for fatty acid methyl esters (FAME) (60 m × 0.25 mm × 0.20 µm; Phenomenex, Torrance, CA, USA). The temperature program involved initiating at 160 °C for 2 min and then incrementally raising by 2 °C per minute until reaching 250 °C; the injector and detector temperatures were both set at 250 °C. The injection volume was in the range of 0.9–1 µL. Identification of peaks was accomplished by comparing them with the known standard mixture of Fas from Sigma-Aldrich (St. Louis, MO, USA), detected by retention times and compared with profiles of chromatograms in the certificate of analysis of the standard mix. The calibration curve was plotted within a working range of 0.156 and 5.0 mg/mL. The results were expressed as a percentage of the total identified FAs. 

### 2.5. Preparation of the Microalgae Extract 

A fresh ChA sample was lyophilized, and a double extraction was performed mixing microalgae powder with 80% (*v*/*v*) methanol/water solution [21]. 

Then, 2.5 mL of 80% methanol was combined with 250 mg of the sample. Following a 2 h stirring period in the dark, the sample underwent centrifugation at room temperature for 30 min at 4000 rpm. The resulting supernatant was collected, while the pellet obtained was subjected to an additional extraction using other 2.5 mL of 80% methanol. The supernatants from both extractions were combined to achieve a final concentration of 50 mg/mL and stored at −20 °C until use. The extraction process was conducted in triplicate.

To assess the preventive efficacy against Ox-LDL-induced damage in HMEC-1 cells, the methanolic extract (50 mg/mL) was concentrated using SpeedVac (Thermo Fisher Scientific, Waltham, MA, USA) until the solvent was completely evaporated, as it exhibited toxicity towards endothelial cells. The resulting sample was subsequently reconstituted in water for achieving 500 mg/mL concentration. Prior to experimentation, the solution underwent filtration using a 0.45 µm filter and, if required, was further diluted with sterile water.

### 2.6. Phenolic Content of ChA Extract

#### 2.6.1. Bioactive Molecules Content 

The total phenols were determined using the Folin–Ciocalteu colorimetric method [22], and the results were expressed as milligrams of gallic acid equivalents (GAE) per 100 g of dry weight (dw). To quantify the total flavonoids, the aluminum chloride colorimetric method was employed, and the findings were presented as milligrams of catechin equivalent (CE) per 100 g of dw [23]. The evaluation of total flavonols was performed following the procedure described by Souid et al. [24], and the results were expressed as milligrams of quercetin equivalent (QE) per 100 g of dw. 

#### 2.6.2. Phenolic Compounds Profiling by UHPLC-ESI-MS/MS

A set of well-known phenolic compounds was selected for the quantitative analysis of the extract using ultra-high-performance liquid chromatography coupled with tandem mass spectrometry (UHPLC-ESI-MS/MS). The analysis was conducted on a Sciex 5500 QTrap+ mass spectrometer (AB Sciex LLC, Framingham, MA, USA), equipped with a Turbo V ion-spray source, and connected to a custom-made ExionLC AC System provided by Shimadzu (Shimadzu Corporation, Kyoto, Japan). The ExionLC AC System consisted of two ExionLC AC Pumps, an Autosampler, a Controller, a Degasser, and a Tray. MS/MS experiments were carried out in the electrospray negative ion mode using nitrogen as the collision gas. The source parameters were set as follows: source type, Turbospray; nebulizer gas (GS1) at 70; turbo gas (GS2) at 50; curtain gas (CUR) at 10; temperature (TEM) at 500 °C; Ionspray Voltage (IS) at −4500 V, and entrance potential (EP) at 10 V. Specific compound parameters, including declustering potential (DP), collision energy (CE), and collision cell exit potential (CXP), were optimized for each Selected Reaction Monitoring (SRM) transition of the targeted components (Table S1, Supplementary Materials). 

Data collection and processing utilized Analyst 1.7.3 software and OS 1.7 software from AB Sciex. Qualitative confirmation was achieved through information dependent acquisition (IDA) criteria, which leveraged the ion trap functionalities of the 5500 + QTrap. This process involved switching from selected reaction monitoring (SRM) to enhanced product ions (EPI) to obtain the MS/MS spectrum using a collision energy (CE) of 35 eV with a CE spread of 15 eV. The obtained spectra were then compared with a custom-made spectral library. Normalization of data was carried out considering the matrix effect and recovery percentage. The matrix effect was calculated as the peak area of the sample spiked after extraction divided by the peak area of the standard, while recovery was calculated as the peak area of the sample spiked before extraction divided by the peak area of the sample spiked after extraction. For quantitative analysis, calibration curves were constructed using a standard mix containing all the analytes at concentrations of 0.5, 1, 2, 4, 8, 16, 32, 64, 128, and 256 ng/mL. 

### 2.7. Antioxidant Activity Assays

The in vitro antioxidant capacity of the ChA extract was investigated using a combination of fluorimetric and spectrophotometric methods. Specifically, the oxygen radical absorbance capacity (ORAC) assay, the ferric reducing antioxidant power (FRAP) assay, and the DPPH radical scavenging assay were employed. To quantify the antioxidant capacity, the ORAC assay described by Ninfali et al. [25] was used. AAPH was utilized as a peroxyl radical generator, and fluorescein served as a probe, while Trolox acted as the standard antioxidant. The results were reported as ORAC units (µmol of Trolox equivalents (TE) per 100 g of dry weight (dw)). For assessing the overall antioxidant power of the ChA extract, the FRAP assay was employed. This colorimetric method, as described by Colosimo et al. [26], relies on the reduction of a ferric tripyridyltriazine complex to its ferrous form. The results were calculated based on the standard curve for Trolox and expressed as µmol of Trolox equivalent (TE) per 100 g of dw. Furthermore, the DPPH radical scavenging activity was determined using the method outlined by Boudjou et al. [27] with some modifications. This anti-radical capacity was expressed as µmol of TE per 100 g of dw.

### 2.8. Endothelial Cell Culture and Treatments

Human microvascular endothelial cells (HMEC-1) were procured from the Centers for Disease Control and Prevention (Atlanta, GA, USA). HMEC-1 cells exhibited morphological, phenotypic, and functional features similar to those of regular human microvascular endothelial cells. The process involved in immortalizing primary cells included transfecting them with a PBR-322-based plasmid that carries the coding region of the Simian virus 40 large T antigen. The cells’ viability was assessed using the Trypan Blue dye exclusion assay, as described by Ades et al. [28]. HMEC-1 were utilized to assess the efficacy of a microalga in reducing endothelial damage caused by oxidized LDL-induced oxidative stress. The cell cultures were established and maintained according to the protocol described by Lubrano and Balzan [29]. To assess the effect of ChA extract on cell viability, the cells were exposed to four different concentrations (6.25, 12.5, 25, and 50 µg/mL) of ChA extract for 24 h. The highest non-toxic concentration on cell viability, which was 25 µg/mL in this case, was selected for the experiment to evaluate the antioxidant capacity against an oxidizing agent, oxidized LDL. Then, 200 µg of apo B/mL of ox-LDL was used to induce oxidative stress. Four sets of cell plates were prepared in triplicate: control (C), oxidized-LDL (Ox-LDL), ChA extract 25 µg/mL (ChA), and Ox-LDL + ChA extract 25 µg/mL (Ox-LDL + ChA). The plates were incubated at 37 °C for 24 h, after which the culture medium was removed for further analysis. The adherent cells were separated by using trypsin/EDTA and then resuspended in type II medium for cell counting using a Burker chamber.

#### 2.8.1. Immunoenzymatic Assays

The levels of LOX-1 (Lectin-Like Oxidized Low-Density Lipoprotein (LDL) Receptor 1) and IL-6 (interleukin-6) in the cell culture medium were measured as indicators of cellular activation and dysfunction. For both LOX-1 and IL-6, the immunoenzymatic assay results were expressed as pg per 100,000 cells (pg/100,000 cells).

The measurements of total nitrite (NO_2_^−^) and nitrate (NO_3_^−^) were conducted using the Griess reaction. The reaction involved the conversion of nitrates to nitrites through the action of nitrate reductase enzyme. Subsequently, the generated nitrites reacted with sulfanilamide, forming a diazotate compound. This compound further reacted with N-(1-naphthyl) ethylenediamine, resulting in the formation of a pink-colored product. The measurement of the product’s absorbance was conducted at 540 nm. All the experiments were carried out three times (triplicate), and the outcomes were presented as micromoles per liter (µmol/L) of NO_2_^−^ + NO_3_^−^ per 100,000 cells (µM NO_2_^−^ + NO_3_^−^/100,000 cells).

#### 2.8.2. RNA Extraction and Real-Time RT-PCR

The isolation of total RNA from HMEC-1 cells (1,500,000 cells/mL) was performed using the E.Z.N.A.^®^ Total RNA Kit I (OMEGA bio-tek, Norcross, GA, USA), then reverse-transcribed by using the iScript™ Advanced cDNA Synthesis Kit (Bio-Rad, Hercules, CA, USA). Quantitative real-time RT-PCR was executed using the SsoFastTMEvaGreen^®^ Supermix (Bio-Rad, Hercules, CA, USA) in a CFX Connect Real-Time PCR Detection System (Bio-Rad, Hercules, CA, USA). Interleukin-6 (IL-6; forward 5′-AAAGCAGCAAAGAGGCAC-3′, reverse 5′-TTCACCAGGCAAGTCTCC-3′), heme oxygenase-1 (HO-1; forward 5′-GCAACAAAGTGCAAGATTCTG-3′, reverse 5′-GCTGAGTGTAAGGACCCAT -3′), NAD(P)H dehydrogenase [quinone] 1 (NQO1), and β-actin (forward 5′-TATCCTGCCGAGTCTGTT-3′, reverse 5′-AGAATGCCACTCTGGAATATC-3′) gene primers were designed using Beacon Designer Software (Premier Biosoft International, San Francisco, CA, USA), and synthesized by Sigma-Aldrich (St. Louis, MO, USA). Each gene was tested three times (triplicate), and the gene expression was determined using the 2^−ΔΔCT^ relative quantification method. The results are presented as the fold change of expression levels compared to the control samples.

### 2.9. Statistical Analysis

Statistical analyses were conducted using XLSTAT Version 2016 statistical software. (Denver, CO, United States) The results are presented as the average value ± standard deviation (SD) and were analyzed using one-way analysis of variance (ANOVA) followed by the Tukey post hoc test. Statistical significance was considered when *p* ≤ 0.05.

## 3. Results and Discussion

### 3.1. Morphological and Molecular Identification 

**Figure 1:**
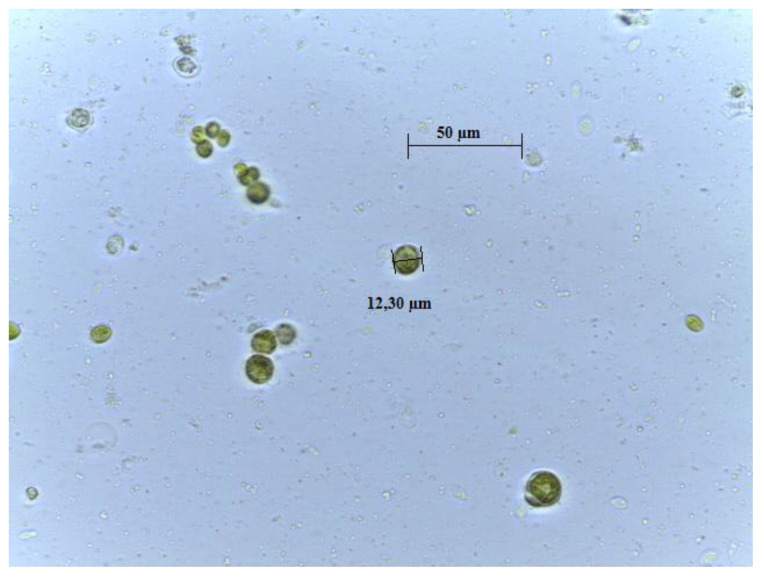
Microscope photography of ChA isolated in laboratory (100×; scale bar 1 cm = 50 µm).

The species was morphologically identified as *Chlamydomonas belonging to Chlamydomonadaceae family* (Figure 1). The molecular characterization provided more trustworthy results to the phylogeny of algae. The PCR amplification of genomic DNA of the microalgal isolates was performed using different primers, i.e., ITS1-ITS4, p23SrV-f1 y p p23SrV-r1. The ITS primers with an annealing temperature of 52 °C determined an amplified fragment of 700 bp. The 23S rRNA gene sequence that was amplified from strains was 410 bp, using primer p23SrV-f1 y p p23SrV-r1 with an annealing temperature of 58 °C [16]. Table 1 displays the specific accession number and the closest identifiable match found in the GenBank nucleotide database. 

### 3.2. Centesimal and Fatty Acid Composition

Table 2 contains the results of the centesimal composition and the fatty acid profile of ChA. The centesimal analysis showed that the microalga is rich in compounds considered of great interest for human nutrition, due to the high content in proteins, lipids, and fibers. Interestingly, the protein content was far higher than those we found in *Ettlia pseudoalveolaris* (13.46 ± 0.12 vs. 8.31 ± 0.12%), another species of green microalga isolated from the same sampling site of Yahuarcocha lake in the Equadorian Highland [31]. The FAs profile revealed a majority of unsaturated ones. A recent study conducted by Khan et al. [32] provided evidence supporting, among other factors, the beneficial impact of the supplementation of omega-3 (ω-3) polyunsaturated fatty acids (PUFAs) in mitigating the risk of cardiovascular disease (CVD). Seafood, specifically oily fish, is well known for exerting beneficial effect on human well-being since it is a significant source of PUFAs; these PUFAs are derived from microalgae, which belong to the phytoplankton community, thus representing the most important part of aquatic ecosystems [33]. Among the diverse fields of application, interestingly, a very recent study by Zaman et al. [34] has demonstrated that that PUFAs extracted from microalgae can be used as a supplement for the chicken patties to prevent lipid oxidation in the product. In particular, ChA results reached in α-linolenic acid (ALA) and linoleic acid (LA) (32.49 ± 0.19 and 17.07 ± 0.09%, respectively), two of the most important PUFAs for human nutrition, together constituted about 50% of the total FAs of this microalga (Table 2). They belong to ω-3 FAs, known to have anti-inflammatory, antibiotic, antiproliferative, antiatherosclerotic, and antithrombotic properties [35]. A previous study by Soares et al. [36] aimed to characterize the FAs profile of various different species of microalgae, among which was *Chlamydomonas planctogloea*, of the same genus of ChA, showed much lower levels of PUFAs. Although slightly lower, we found comparable levels of ALA and LA (29.28 ± 0.12 and 14.95 ± 0.06, respectively) in the green microalga *Ettlia pseudoalveolaris* [31]. Nevertheless, ChA also exhibited considerable quantities of saturated fatty acids (SFAs), particularly palmitic acid (C16:0), which accounted for the majority (39.46 ± 0.25%) of the composition (Table 2). Albeit in smaller quantities, additional SFAs, such as myristic acid (C14:0) and stearic acid (C18:0), were also present and possess diverse industrial applications. For instance, the abundance of palmitic and stearic acids is desirable in high-quality biodiesel production, as a higher proportion of SFAs contributes to improve viscosity (flow properties) and enables the utilization of biodiesel in low-temperature environments [37]. Notably, a recent analysis of the FA profile of *Chlorella vulgaris* across four distinct strains and of *Ettlia pseudoalveolaris* highlighted palmitic acid as the predominant SFA [31,38]. Moreover, investigations on other green microalgal strains, namely *T. obliquus* and *D. abundans*, showed the prevalence of SFAs as the primary constituents in their total fatty acid methyl esters profiles, reaffirming the richness of this FA type in numerous microalgae [39].

It Is important to take into account that the values of the centesimal composition of microalgae can vary within the same species based on the cultivation method, collection timing, and environmental stress factors. Factors like the presence or absence of nutrients can significantly influence these variations [36,40].

### 3.3. Bioactive Compounds Quantitation and Antioxidant Activity of ChA Extract

The freeze-dried ChA methanolic extract displayed the following content of bioactive compounds: total phenols, 177.55 ± 25.94 mg GAE/100 g dw; flavonoids, 440.15 ± 152.38 mg CE/100 g dw; flavonols, 203.80 ± 97.02 mg QE/100 g dw (Table 3). To ensure meaningful comparisons with extracts from other green microalgae, it is crucial to take into account that the antioxidant composition is greatly influenced by various factors. These factors include the growth conditions of the microalgae, such as nutrient availability, temperature, and stress factors. Additionally, the choice of solvents used during extraction and, of course, the specific species being studied also play a significant role in determining the antioxidant profile. [41,42]. It is interesting to note that in a recent study by Faraloni et al. [43], *Chlamydomonas reinhardtii*, a green alga of the same genus of ChA, exhibited an enhanced production of polyphenols when exposed to high levels of light, thus indicating that light exposure levels also can influence total phenols content. The polyphenolic content of our extract from ChA was similar to those obtained by Goiris et al. [41], who examined biomass from 32 microalgal species and reported an average total polyphenol content of 211 mg GAE/100 g dw. In another study, Santhakumaran et al. [44] analyzed the total phenols content of 25 green microalgal species extracted with different solvent, with values ranging from 0 to 2820 mg GAE/100 g. Results obtained for ChA are also concordant with those found for other algae of the genus *Chlamydomonas,* varying from 60 to 1506 mg GAE/100 g [45,46]. Currently, the literature offers limited studies focusing on specific polyphenol subcategories [8]. These studies confirm that the quantity of these compounds can vary significantly depending on the species and growth conditions [47]. The high variability in phenolic contents is also within the same species of microalga, and it is not explainable with microalgal phylogenetic clusters [48]. Growing conditions such as growing medium, pH, temperature, and light intensity exposure contribute to the natural differences in bioactive substance content [49,50,51]. Inizio modulo Dinev et al. [52] investigated the total flavonoid content of methanolic extracts of four different microalgae strains, which resulted to be in the range of 229  ±  0.19 up to 668  ±  0.3 mg CE/100 g, in accordance with our findings. In another study conducted by Tiong et al. [53], the concentration of flavonoids was measured in five different microalgal species, revealing a range from 1400 to 3470 mg of CE/100 g dw, which are higher values than those we observed in our study. The antioxidant potential of the ChA extract was evaluated using three in vitro assays, which assessed the antioxidant capacity (ORAC), the radical scavenging activity (DPPH), and the metal-related antioxidant power (FRAP). Our results showed that the microalgae extract exhibited a good antioxidant activity, with an ORAC value of 4827.66 ± 1.33 µmol TE/100 g dw, a value comparable to those found by Banskota et al. [54] for nine different species of microalgae, ranging from 2121 to 6948 µmol TE/100 g. The antioxidant capacity was further assessed spectrophotometrically using the FRAP and DPPH assays, which were selected to explore different mechanisms of antioxidant activity in the samples. Specifically, the FRAP assay measures the microalgal strains’ ability to reduce ferric-tripyridyltriazine (Fe^3+^-TPTZ) to a blue-colored ferrous-tripyridyltriazine complex (Fe^2+^-TPTZ) under low pH conditions. On the other hand, the DPPH assay is based on the reduction in the violet-colored DPPH radical to a pale-yellow-colored molecule by donating an electron or hydrogen atom [55]. Together with the results of ORAC assay, a FRAP value of 45.53 ± 5.53 and a DPPH antiradical activity of 375.51 ± 3.62 µmol TE/100 g dw suggested a potential role in preventing oxidative damage to biomolecules (Table 3).

### 3.4. Phenolic Compounds Profiling by UHPLC-ESI-MS/MS

The UHPLC-ESI-MS/MS phenolic compounds profile of the methanol/water (80%) extract revealed a wide range of phenol derivates in ChA (Table 4). We detected traces of phenolic acids and flavones, a good amount of flavonols, in particular, quercetin, and flavan-3-ols, in particular, epicatechin. However, the most present compound we found in the extract by far was verbascoside (158.17 ± 16.67 out of a total of 169.14 µg/g dw), a caffeoyl phenylethanoid glycoside with well-known antimicrobial properties through modulating protein synthesis and impeding leucine assimilation [56]. Up to now, in the literature, the comprehensive examination of phenolic compounds in microalgae using HPLC remains insufficient, thus hindering the comparison of our findings. Moreover, the existing literature lacks a thorough elucidation of the phenolic composition specific to this particular genus of microalga. Among the few available studies, in a recent one by Faraloni et al. [43], the green microalga *Chlamydomonas reinhartii* showed lower levels of phenolic compounds, especially of chlorogenic, coumaric, and gallic acid, compared to those we found in ChA. Goiris et al. [57] studied simple phenols and flavonoids in different microalgal biomass samples and detected p-coumaric, ferulic, and caffeic acid, quercetin, phloretin, apigenin, kaempferol, and catechin but to a lesser extent. Microalgae are considered a natural source of pigments with regard to chlorophyll, carotenoids, and phycobiliproteins [58]. Less importance is given to the class of pigments anthocyanidin because their presence in microalgae is reported as uncertain [59]. Only the study by Tanna et al. [60] reported cyanidin and malvidin derivatives in the green alga *Caulerpa racemosa.* Herein, for the first time, five anthocyanins, albeit in small quantities, were identified (Table 4). Verbascoside, the most abundant phenolic compound of ChA extract, is one of the most frequently reported in all algal species worldwide [8]. Several studies demonstrated the role of verbascoside in the prevention and treatment of various human diseases and disorders due to its anti-inflammatory and antioxidant activities [61,62,63]. In our previous investigation focusing on the green microalga *E. pseudoalveolaris*, we observed a prominent presence of verbascoside, similar to ChA, among the identified phenolic acids. Notably, the extract of this microalga exhibited a substantial antioxidant capacity and a noteworthy inhibition of growth in various Gram-positive and Gram-negative bacterial strains, which is likely attributed to the presence of this derivative of caffeic acid [31]. Further investigation would be necessary to assess whether verbascoside and the other detected phenols confer to ChA this antibacterial capacity observed for *E. pseudoalveolaris*. It is imperative to acknowledge that the efficiency of polyphenol extraction, as well as its concentration, is contingent upon the polarity of the solvent employed, as it governs the solubility of polyphenols based on their structural properties. Therefore, the reliability of these findings largely depends on the specific methodology used.

### 3.5. Effects on HMEC-1 Cells Treated with Ox-LDL

**Figure 2:**
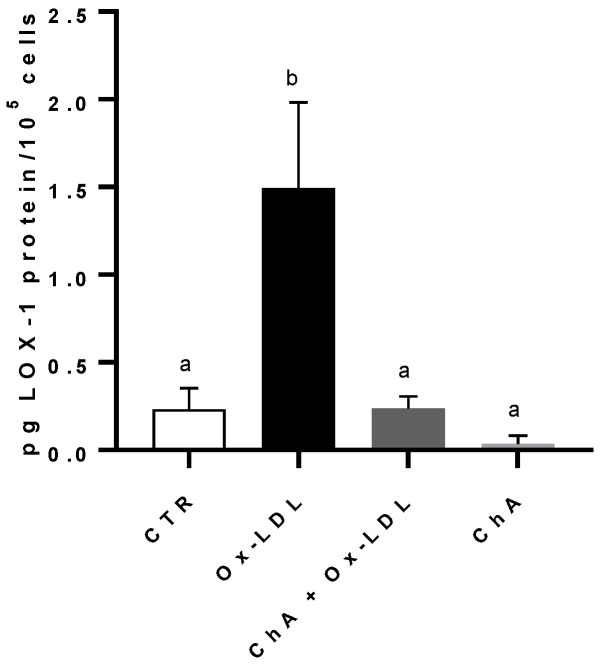
LOX-1 protein levels in HMEC-1 cells by immunoenzymatic assay, expressed as pg of LOX-1 per 100,000 cells, measured in CTR, Ox-LDL, Ox-LDL + ChA (*C. agloeformis*), and ChA cells. Experiment was carried out in triplicate and data represent the mean ± SD (bars). a, b: values significantly different by one way ANOVA-test, *p* ≤ 0.05.

Compared to the control, the incubation of HMEC-1 cells with Ox-LDL determined a significant increase in LOX-1 protein level (respectively, 0.23 ± 0.12 pg of LOX-1/10^5^ cells and 1.49 ± 0.49 pg of LOX-1/10^5^ cells). When we treated the cells with both ChA and Ox-LDL, we did not observe any significant increase in LOX-1 expression, which remained similar to the control cells (0.24 ± 0.07 pg/10^5^ cells). This was in contrast to the treatment with ChA alone, where LOX-1 expression was significantly lower (0.04 ± 0.05 pg/10^5^ cells). (Figure 2).

**Figure 3:**
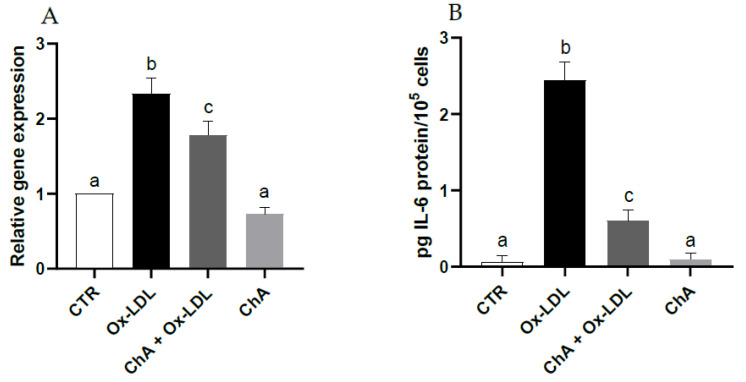
IL-6 relative gene expression by real-time RT-PCR (**A**) and protein levels by immunoenzymatic assay (**B**) expressed as pg of IL-6 per 100,000 cells, in HMEC-1 cells measured in CTR, Ox-LDL, Ox-LDL + ChA (*C. agloeformis)* and ChA cells. Experiment was carried out in triplicate and data represent the mean ± SD (bars). Results of relative gene expression are normalized for the levels of housekeeping gene β-actin and referred to the mean of the controls, to which a value of 1 was assigned. a, b, c: values significantly different by one way ANOVA-test, *p* ≤ 0.05.

With respect to control cells, those exposed to Ox-LDL exhibited a significant increase in IL-6 gene expression (about 2.3-fold increase vs. CTR) and protein levels (0.06 ± 0.08 pg/10^5^ and 2.45 ± 0.24 pg/10^5^ cells, respectively). When the HMEC-1 cells were exposed to both ChA and Ox-LDL, the IL-6 levels decreased significantly at both transcriptional (about 1.8-fold increase vs. CTR) and protein level (0.61 ± 0.14 pg/10^5^ cells) compared with cells incubated only with Ox-LDL, albeit remaining significantly higher than the control. Treatment with ChA alone showed no significant change with respect to control cells at transcriptional and protein levels (0.10 ± 0.08 pg/10^5^ cells) (Figure 3A,B).

High levels of LDL-cholesterol are considered a risk factor for various diseases; it is well established that the effect of oxidative modification of LDL is the real trigger for diseases such as cardiovascular disease [64]. LOX-1 is an important scavenger receptor, first identified by Sawamura and collaborators [65]. It is very abundant on the surface of vascular endothelium and is able to bind and internalize Ox-LDL through endocytosis [66]. Many studies have shown that the cellular internalization of Ox-LDL is an important cause for endothelial activation and dysfunction that arise in atherogenesis [67]. In addition, the binding of Ox-LDL to LOX-1 promotes a significant increase in reactive oxygen species (ROS) generation [68], activating cellular biochemical pathways such as the Nuclear Factor-kB (NF-kB) pathway with the production of inflammatory cytokines [69]. In this study, HMEC-1 exposition to Ox-LDL was exploited to investigate the potential contribution of ChA in protecting the endothelium. Our study demonstrated that LOX-1 and IL-6 were basically produced by HMEC-1 cells. The presence of Ox-LDL significantly increased LOX-1 and IL-6 levels, which were instead neutralized by the concurrent incubation with ChA, bringing LOX-1 concentrations back to control levels and reducing IL-6 levels (Figure 2 and Figure 3). However, IL-6 levels remained significantly different from those of the control, at both transcriptional and protein level; probably, additional hours of exposure might have been needed. In fact, while LOX-1 is directly modulated by Ox-LDL, IL-6 levels are increased by other biochemical pathways like the NF-kB pathway [70], thus probably leading to a delay in reducing this cytokine. The available evidence regarding the influence of diet on microvascular function is limited [71]. Adverse effects on microcirculation can be mitigated through dietary restriction or appropriate supplementation [72]. Dietary patterns characterized by the consumption of food sources abundant in polyphenols have demonstrated potential direct or indirect beneficial effects on microvascular function, both in healthy individuals and those with cardiovascular disease (CVD) [73,74]. Furthermore, it is well established that the consumption of food rich in ω-3 polyunsaturated fatty acids PUFAs exerts a positive influence on microcirculation.

Our results showed that polyphenols- and PUFAs-rich ChA extract has a protective effect on microvascular function, which exerts through a direct action on Ox-LDLs that may be due to its antioxidant capacity that inhibit the binding of ox-LDL to the receptor. Moreover, the study demonstrated that ChA was not toxic toward endothelial cells.

**Figure 4:**
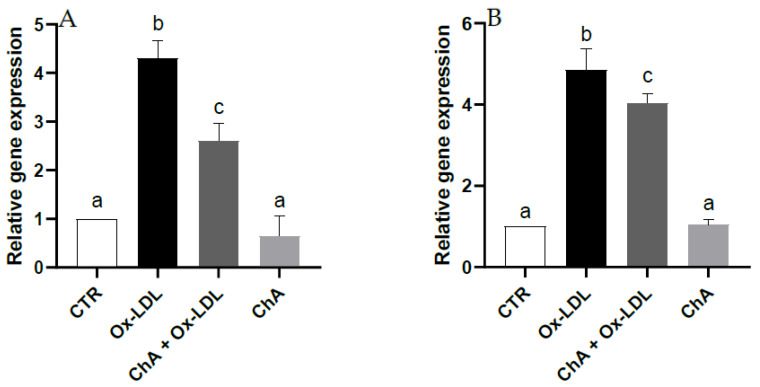
Relative gene expression of HO-1 (**A**) and NQO1 (**B**) in HMEC-1 cells measured by real-time RT-PCR in CTR, Ox-LDL, Ox-LDL + ChA (*C. agloeformis*) and ChA cells. Experiment was carried out in triplicate and data represent the mean ± SD (bars). Results of relative gene expression are normalized for the levels of housekeeping gene β-actin and referred to the mean of the controls, to which a value of 1 was assigned. a, b, c: values significantly different by one way ANOVA-test, *p* ≤ 0.05.

Quantitative real time RT-PCR demonstrated a notable increase in the gene expression of HO-1 and NQO1 (about 4.3- and 4.9-fold increase vs. CTR, respectively) following Ox-LDL treatment. Upregulation of HO-1 and NQO1 was significantly inhibited (about 2.6- and 4-fold increase vs. CTR, respectively) by pre-treatment with the ChA extract. Furthermore, no significant change was observed for cells exposed to ChA alone compared to the control (Figure 4). 

The effect of Ox-LDL on nuclear factor erythroid 2-related factor (Nrf2), a transcription factor involved in the induction of antioxidant enzymes and molecules which acts as radical scavengers was investigated by analyzing the gene expression of HO-1 and NQO1 [75]. Ox-LDL exposure increased their transcription by Nrf2-mediated antioxidant endogenous response to ROS generation [76]. We might have expected that the co-exposure with ChA determined a further increase in the antioxidant response to Ox-LDL treatment. In fact, phenolic compounds, of which ChA extract is rich, exert their antioxidant protective function also by activating the transcription factor Nrf2, which, in turn, upregulates the expression of the two genes. In our experimental conditions, it is likely that HO-1 and NQO1 upregulation is no longer needed due to the decrease in pro-oxidant LOX-1 and IL-6 induced by ChA treatment. In fact, as mentioned before, the phenolic compounds rich-extract may have determined the inhibition of the binding of Ox-LDL to the receptor LOX-1 and the reduction in the pro-inflammatory cytokine by inhibiting NF-kB that mediates inflammatory processes.

**Figure 5:**
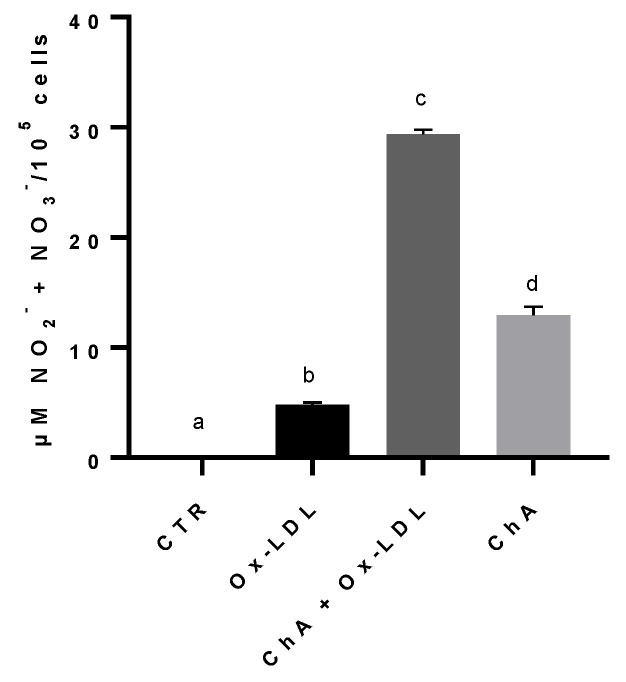
Nitrites and nitrates levels in HMEC-1 cells based on Griess reaction, expressed as µM NO_2_^−^ + NO_3_^−^/100,000 cells, measured in CTR, Ox-LDL, Ox-LDL + ChA (*C. agloeformis*) and ChA cells. Experiment was carried out in triplicate and data represent the mean ± SD (bars). a, b, c, d: values significantly different by one-way ANOVA-test, *p* ≤ 0.05.

The NO metabolites, nitrites and nitrates, content increased significantly in cells treated with Ox-LDL (4.81 ± 0.19 µM NO_2_^−^ + NO_3_^−^/10^5^ cells), compared to the control (0.03 ± 0.04 µM/10^5^ cells). The exposure of HMEC-1 cells to both ChA and Ox-LDL resulted in an even greater increase (29.37 ± 0.40 µM/10^5^ cells), statistically significant in comparison to both control cells and Ox-LDL-treated cells. The level of nitrites and nitrates in cells treated with ChA alone (12.96 ± 0.74 µM/10^5^ cells) was significantly higher in comparison to the control and cells treated with Ox-LDL, but significantly lower than ChA + Ox-LDL group (Figure 5).

A different trend was observed for the production of nitrites and nitrates with respect to those of LOX-1 and IL-6. The increase in NO metabolites is related to vasodilation. In general, the presence of many free oxygen radicals tends to reduce the levels of NO by transforming it in peroxynitrite and to damage the endothelial nitric oxide synthase (eNOS) as well as to oxidize tetrahydrobiopterin to dihydrobiopterin leading to eNOS uncoupling [77]. Our results showed that the treatment with Ox-LDL significantly increased nitrites and nitrates production compared to controls, mimicking a beneficial effect. It is likely that the presence of Ox-LDL induces the eNOS by stimulating an inflammatory process, thus producing large amounts of NO as a compensatory mechanism. The initial reduction in NO caused by oxidative stress could be the stimulus that drives eNOS to increase NO production which, then, we observe in the form of nitrites/nitrates [78]. A synergistic effect was observed when cells were treated with Ox-LDL and ChA, with both treatments driving eNOS to produce NO. The treatment with ChA alone determined a beneficial effect on eNOS by a significant increase in NO metabolites production, compared to controls. Although there are no data in the literature, our previous unpublished studies also demonstrated the presence of an inducible form of the enzyme (iNOS) in endothelial cells, which would provide further explanation for the trend of NO production during the Ox-LDL-induced inflammatory process.

## 4. Conclusions

Microalgae are recognized as an exceptional, yet underexplored, natural resource for a healthy diet. The absence of polysaccharides within their cellular structure renders their biomass a readily digestible substance, thereby enhancing its suitability for human consumption. To the best of our current knowledge, this investigation represents the first study aimed at elucidating the fatty acids and phenolic compounds composition, as well as evaluating the extract’s antioxidant activity of the green microalgae isolated from freshwater highland lakes of Ecuador and identified as *Chlamydomonas agloeformis*. The present study revealed that ChA represents a promising source of high-quality nutrients and bioactive compounds that make it a promising functional food. Particularly, FAs analysis revealed that this microalga exhibits a notable abundance of PUFAs, specifically ALA and LA. These PUFAs are of great significance for human health, as they are essential omega-3 FAs that must be obtained through dietary sources in order to maintain a balanced intake. Our findings demonstrated that ChA possesses a substantial quantity of phenolic compounds known to play a pivotal role in determining the observed antioxidant activity of the extract. The microvascular endothelial cells, HMEC-1, represent a basic in vitro experimental model to validate the protective effect of the extract derived from the bioactive-substances-rich green microalga on the endothelium. Notably, the application of ChA resulted in a significant decrease in LOX-1 and IL-6 expression and an increase in NO production in response to the oxidative stress induced by Ox-LDL treatment of HMEC-1. Antioxidants are widely acknowledged for their advantageous effects on health and their pivotal role in shielding cells against the detrimental consequences of free radicals. Given the escalating demand for these value-added products, microalgae undeniably offer a compelling solution since their antioxidant capacity matches or surpasses that of higher plants or fruits. 

## Figures and Tables

**Table 1:** Comparisons of amplified sequences base on GenBank Information. Molecular identification was performed twice.

Sequency	Taxonomy	Identity (%)	Sequency GenBank (NCBI)	References
Chla	*Chlamydomonas agloeformis*	99.5	L43351.1	[30]

**Table 2:** Composition expressed as fraction on dry weight (% DW), and fatty acids (fAs) profile expressed as % of identified fatty acids of *Chlamydomonas agloeformis*.

Reference Compounds	ChA
Moisture	50.25 ± 0.03
Ashes	28.15 ± 0.04
Lipids	1.21 ± 0.11
Protein	13.46 ± 0.12
Crude Fiber	6.93 ± 0.01
**SFAs**	
C14:0	1.45 ± 0.03
C15:0	0.51 ± 0.01
C16:0	39.46 ± 0.25
C18:0	1.74 ± 0.02
C20:0	0.74 ± 0.00
**MUFAs**	
C14:1	0.38 ± 0.01
C16:1-n7	2.85 ± 0.03
C18:1-n9	1.53 ± 0.02
**PUFAs**	
C18:2-n6 (LA)	17.07 ± 0.09
C18:3-n3 (ALA)	32.49 ± 0.19
C20:4 (AA)	0.95 ± 0.03
C22:5-n3 (DPA)	0.83 ± 0.01

SFAs: saturated fAs; MUFAs: monounsaturated fAs; PUFAs: polyunsaturated fAs. Values are reported as means of three replicates ± SD.

**Table 3:** Bioactive compounds and in vitro antioxidant activity of ChA methanolic extract.

		ChA
**Bioactive compounds**	Total phenols (mg GAE/100 g dw)	177.55 ± 25.94
	Flavonoids (mg CE/100 g dw)	440.15 ± 152.38
	Flavonols (mg QE/100 g dw)	203.80 ± 97.02
		
**Antioxidant activity**	ORAC (µmol TE/100 g dw)	4827.66 ±1.33
	FRAP (µmol TE/100 g dw)	45.53 ± 5.53
	DPPH (µmol TE/100 g dw)	375.51 ± 3.6

Values are reported as means of three replicates ± SD.

**Table 4:** Content of individual phenolic compounds of extract from ChA (µg/g dw).

Phenolic Compound	Concentration (µg/g dw)
Gallic Acid	1.81 ± 0.01
Cyanidin 3,5-*O*-diglucoside	0.06 ± 0.00
3-*O*-Caffeoylquinic acid	1.64 ± 0.05
Caffeic Acid	1.82 ± 0.02
Vanillic Acid	1.33 ± 0.10
4-Coumaric Acid	0.49 ± 0.01
*trans*-Ferulic Acid	0.12 ± 0.04
Rosmarinic Acid	0.43 ± 0.18
∑ Phenolic acids *	**7.69**
Quercetin	1.99 ± 0.33
Quercetin 3-O-glucoside	46.14 ± 8.13
Quercetin 3,4-O-diglucoside	0.83 ± 0.03
Quercetin 3-O-rutinoside	1.65 ± 0.33
Kaempferol 7-*O*-glucoside	1.02 ± 0.12
Kaempferol 3-*O*-glucoside	0.31 ± 0.19
Kaempferol 3-*O*-rutinoside	0.38 ± 0.05
∑ Flavonols *	**52.32**
Luteolin	0.34 ± 0.06
∑ Flavones *	**0.34**
Apigenin	0.02 ± 0.01
(+)-Catechin	1.71 ± 0.09
(−)-Epicatechin	29.40 ± 2.51
∑ Flavan-3-ols *	**31.14**
Hydroxytyrosol	0.34 ± 0.02
Verbascoside	158.17 ± 16.67
Oleuropein	3.08 ± 0.00
Ligstroside	0.40 ± 0.00
Phloridzin	0.02 ± 0.00
Phloretin	7.06 ± 0.10
Resveratrol 3-*O*-glucoside	0.03 ± 0.01
Naringenin	0.03 ± 0.02
∑ Others *	**169.14**

Values are reported as means ± SD. * Sum of phenolic acids, flavonols, flavones, flavan-3-ols and other compounds determined by HPLC-MS

## Data Availability

Data are contained within the article. The data presented in this study are available in the present article.

## Supplementary Materials

The following supporting information can be downloaded at: https://www.mdpi.com/article/10.3390/foods12173147/s1, Table S1: The retention times (t_R_), selected reaction monitoring (SRM) transitions and relative MS/MS parameters of phenolic compounds detected in extract from ChA.
