# Supplementary material for: Chlamydomonas agloeformis from the Ecuadorian Highlands: Nutrients and Bioactive Compounds Profiling and In Vitro Antioxidant Activity
Source: Foods. 2023 Aug 22;12(17):3147. doi: 10.3390/foods12173147 (PMC10487033; doi:10.3390/foods12173147)
Supplement: Supplementary file 1 [file foods-12-03147-s001.zip › foods-2510068-supplementary.pdf]

foods

MDPI

Article

Chlamydomonas agloeformis from the Ecuadorian Highlands: Nutrients and Bioactive Compounds Profiling and In Vitro Antioxidant Activity

Teresa Grande [1,2,†], Andrea Vornoli [1,†], Valter Lubrano [3], Francesco Vizzarri [4], Andrea Raffaelli [1,5], Morena Gabriele [1], Jeniffer Novoa [6], Carla Sandoval [6], Vincenzo Longo [1], Maria Cristina Echeverria [6] and Luisa Pozzo [1,*]

[1] Institute of Agricultural Biology and Biotechnology-National Research Council (IBBA-CNR), Via Moruzzi 1, 56124 Pisa, Italy; teresa.grande@unifi.it (T.G.); andrea.vornoli@ibba.cnr.it (A.V.); andrea1.raffaelli@santannapisa.it (A.R.); morena.gabriele@ibba.cnr.it (M.G.); vincenzo.longo@ibba.cnr.it (V.L.)
[2] Department of Experimental and Clinical Biomedical Sciences "Mario Serio", University of Florence, Viale Morgagni 50, 50134 Florence, Italy
[3] Fondazione G. Monasterio, CNR/Regione Toscana, 56124 Pisa, Italy; walterl@ftgm.it
[4] National Agricultural and Food Centre Nitra, Hlohovecká 2, 95141 Lužianky, Slovakia; francesco.vizzarri@nppc.sk
[5] Crop Science Research Center, Scuola Superiore Sant'Anna, Piazza Martiri della Libertà 33, 56127 Pisa, Italy
[6] eCIER Research Group, Department of Biotechnology, Universidad Técnica del Norte, Av. 17 de Julio 5–21 y Gral. José María Córdova, Ibarra 100150, Ecuador; jpnovoar@utn.edu.ec (J.N.); casandoval@utn.edu.ec (C.S.); mecheverria@utn.edu.ec (M.C.E.)
* Correspondence: luisa.pozzo@ibba.cnr.it
† These authors contributed equally to this work.

Table S1. The retention times (t_R), selected reaction monitoring (SRM) transitions and relative MS/MS parameters of phenolic compounds detected in extract from ChA.

No.	Compound Name	Phenolic Class	t_R (min)	Q1	Q3	DP (V)	CE (eV)	CXP (V)
1	Gallic acid	Phenolic acid	1.28	168.9	125.0	−75	−20	−13
2	Hydroxytyrosol	Phenylethanoid	1.92	153.0	123.0	−100	−20	−17
3	Cyanidin 3,5-*O*-diglucoside (cyanin)	Anthocyanin	2.12	611.1	287.1	142	42	14
4	3-*O*-Caffeoylquinic acid (chlorogenic acid)	Phenolic acid	2.56	353.0	191.0	−61	−24	−9
5	(+)-Catechin	Flavan-3-ol	2.69	289.0	244.9	−108	−22	−11
6	Caffeic acid	Phenolic acid	2.80	178.9	135.0	−86	−23	−11
7	Vanillic acid	Phenolic acid	2.86	166.9	108.0	−62	−26	−13
8	(−)-Epicatechin	Flavan-3-ol	2.98	289.0	244.9	−108	−22	−11
9	Quercetin 3,4-*O*-diglucoside	Flavonol	3.00	625.1	270.9	−178	−85	−12
10	Quercetin 3-*O*-rutinoside (rutin)	Flavonol	3.29	609.2	299.9	−154	−48	−11
11	4-Coumaric Acid	Phenolic acid	3.35	163.0	119.0	-65	-48	-11
12	Quercetin 3-*O*-glucoside	Flavonol	3.46	463.1	300.0	−154	−37	−5
13	Verbascoside	Phenylethanoid	3.48	623.1	160.9	−82	−43	−7
14	Kaempferol 3-*O*-rutinoside	Flavonol	3.52	593.2	284.9	−138	−40	−5
15	Resveratrol 3-*O*-glucoside (piceid)	Stilbenoid	3.54	389.1	227.0	−125	−32	−11
16	*trans*-Ferulic acid	Phenolic acid	3.65	193.0	134.0	−62	−20	−8
17	Kaempferol 7-*O*-glucoside	Flavonol	3.69	447.1	284.9	−158	−38	−5
18	Kaempferol 3-*O*-glucoside	Flavonol	3.85	447.1	284.1	−202	−39	−11
19	Rosmarinic Acid	Phenolic acid	3.97	359.0	161.0	--70	-22.5	-10

20	Phloridzin	Dihydrochalcone	4.01	435.1	272.9	−135	−23	−5
21	Oleuropein	Secoiridoid	4.09	539.1	275.0	−137	−32	−12
22	Ligstroside	Secoiridoid	4.42	523.1	291.0	−117	−32	−11.8
23	Luteolin	Flavone	4.60	284.9	133.0	−130	−44.6	−17.4
24	Quercetin	Flavonol	4.64	301.0	150.9	−113	−38	−8
25	Naringenin	Flavanone	5.02	270.9	150.9	−120	−25	−10.5
26	Apigenin	Flavan-3-ol	5.04	268.9	117	-120	-49	-14
27	Phloretin	Dihydrochalcone	5.13	273.0	167.0	−103	−38	−11
